# Clipless laparoscopic cholecystectomy is a better technique in reducing intraoperative bleeding

**DOI:** 10.1016/j.amsu.2021.01.039

**Published:** 2021-01-30

**Authors:** Sabry Abounozha, Talal Alshahri, Samer Alammari, Rashid Ibrahim

**Affiliations:** a Northumbria Healthcare NHS Foundation Trust, Northumbria, UK; b Imam Abdulrahman Alfaisal University Hospital, Riyadh, Saudi Arabia; c King Khaled Hospital, Najran, Saudi Arabia; d University Hospitals Plymouth NHS Trust, Plymouth, UK

**Keywords:** Clipless cholecystectomy, Conventional cholecystectomy, Standard cholecystectomy, Vessel sealing devices, Ultrasonic scalpel, Blood loss, Bleeding

## Abstract

A best evidence topic has been constructed using a described protocol. The three-part question addressed was: In patients undergoing cholecystectomy is the clipless laparoscopic cholecystectomy associated with lower rates of intraoperative bleeding compared to conventional cholecystectomy?

The search has been devised and 5 studies were deemed to be suitable to answer the question. The outcome assessed was the rate intraoperative blood loss in clipless cholecystectomy compared to conventional laparoscopic cholecystectomy. Authors recommend adopting clipless laparoscopic cholecystectomy especially in patients with high risk of intraoperative bleeding.

## Highlights

• Perioperative Bleeding with laparoscopic cholecystectomy can be an troublesome especially in high risk patients.
• Clipless laparoscopic cholecystectomy entails using energy device to dissect and to seal the cystic duct and artery.
• Clipless cholecystectomy can offer a sensible solution for this particular situations.

## 1 Introduction

This BET was constructed using a framework outlined by the International Journal of Surgery [1]. A BET provides evidence-based answers to common clinical questions, using a systematic approach of reviewing the literature.

## 2 Clinical scenario

You are going to perform a laparoscopic cholecystectomy in a patient with anticipated high tendency of intraoperative bleeding especially during the dissection of the gallbladder liver bed. You are thinking about the best technique to decrease this risk. Therefore, you decide to conduct a systematic review to look for a based evidence answer to this question.

## 3 Three-part question

In [patients undergoing cholecystectomy] is [the clipless laparoscopic cholecystectomy] associated with [lower rates of intraoperative bleeding compared to conventional cholecystectomy]?

## 4 Search strategy

The search was conducted as following:

Embase 1974 to 2020 and MEDLINE® 1946 to November 2020 using the OVID interface. [clipless cholecystectomy OR ultrasonic cholecystectomy OR vessel sealing device cholecystectomy] AND [conventional cholecystectomy OR standard cholecystectomy] AND [intraoperative bleeding OR blood loss]

The search was limited to English language and human studies.

## 5 Search outcome

**Fig. 1:**
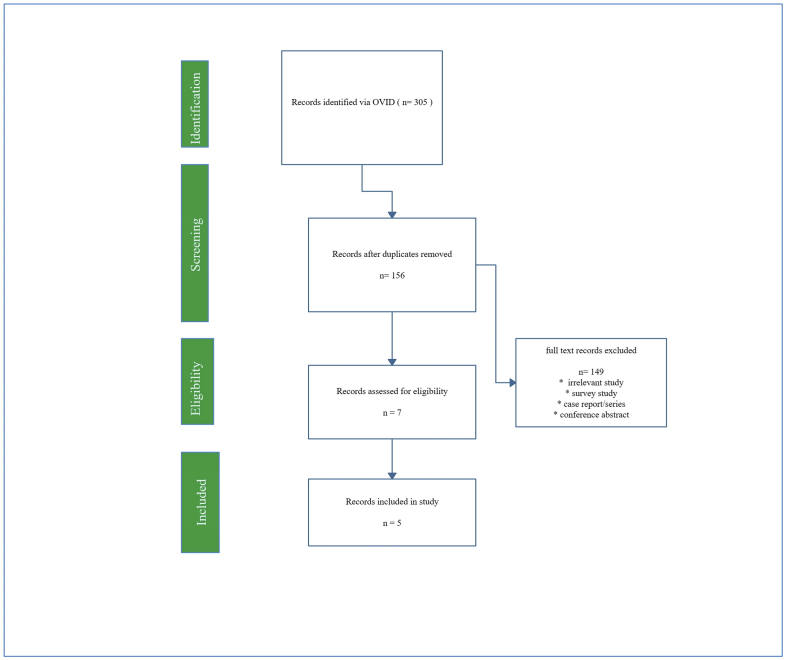
PRISMA Flow Chart.

305 articles were found. Out of these 5 deemed to be suitable and met the criteria of our search after removing the duplicate and excluding the irrelevant articles Fig. 1.

Exclusion criteria:

1 Studies not comparing both techniques
2 Conference abstracts
3 Low evidence papers
4 Absence of full-text articles

## 6 Result

(please refer to the table) Table 1

**Table 1:** Evidence-based answers to common clinical questions.

Author, date of publication, journal and country	Study type and level of evidence	Patient group	Outcomes	Follow-up	Key results	Additional comments
Kandil et al. [2], 2009, J Gastrointest Surg, Egypt	Randomized Controlled Study, Level II	140 patients. group A included 70 patients in whom LC was conducted using the traditional method (TM) by clipping both cystic duct and artery and dissection of gallbladder from liver bed by diathermy, and group B (70 patients) LC was conducted using harmonic scalpel (HS) closure and division of both cystic duct and artery and dissection of gallbladder from liver bed by HS	to compare the traditional method of laparoscopic cholecystectomy (LC) versus LC using harmonic as regard to intraoperative blood loss	Both groups were followed-up for 6 months	HS provides a complete haemostasis and is a safe alternative to stander clip of cystic duct and artery. Intraoperative blood loss was significantly more in the traditional group than in the HS group (83.31 + 46.23 vs. 43.28 + 31.27; p = 0.0001)	Single Centre, randomized, no power calculation, no blinding was mentioned, patients above 80 years old, patients with history of upper laparotomy, patients with common bile duct stones and pregnant women were excluded, risk of bias cannot be excluded
Nakeeb et al. [3], 2010, Surg. Endoscopy Journal, Egypt	Randomized Controlled Study, Level II	120 patients. group A (60 patients) underwent LC by the traditional method (TM) with clipping of both the cystic duct and artery and dissection of the gallbladder by diathermy, and group B (60 patients) had LC performed using Harmonic scalpel (HS) closure and division of both the cystic duct and artery with dissection of the gallbladder by the HS	This study aimed to compare the traditional method for LC with LC using the Harmonic scalpel in terms of safety and intraoperative blood loss for cirrhotic patients	Both groups were followed-up for 6 months	The Harmonic scalpel provides complete haemostasis and is a safe alternative to the standard clipping of the cystic duct. The intraoperative blood loss was significantly greater in the traditional group than in the HS group (133 ± 131.13 vs. 70.13 ± 80.79 ml; p = 0.002)	Single Centre, randomized, no power calculation, no blinding was mentioned, patients older than 80 years, patients with a history of upper laparotomy, patients with common bile duct stones, patients with decompensated liver disease, and pregnant women were excluded, risk of bias cannot be excluded
Jain et al. [4], 2011, Journal of Laparoendoscopic & Advanced Surgical Techniques, India	Randomized Controlled Study, Level II	200 patients with symptomatic gallstone disease, randomly divided into two groups (100 each), one undergoing cholecystectomy using ultrasonically activated shears and the other using conventional clip and electrocautery	to compare the traditional method of laparoscopic cholecystectomy (LC) versus LC using harmonic as regard to intraoperative blood loss and postoperative haemoglobin drop	Both groups were followed-up for 6 months	Ultrasonically activated scalpel can be used safely in laparoscopic cholecystectomy without risk of major injuries. There was greater fall in haemoglobin (0.53 versus 1.33 g%; P value of 0.001) and haematocrit (1.59 versus 2.60; P value of 0.001) when electrocautery was used compared to Harmonic scalpel (clipless cholecystectomy)	Single Centre, randomized, randomization process is not clear, no power calculation, no blinding was mentioned, patients above 70 years old, impaired liver function tests, history of jaundice or pancreatitis, suspicion of gallbladder carcinoma, patients having concomitant common bile duct (CBD) calculi, acute cholecystitis, cholangitis, and empyema of gallbladder, pregnant patient, CBD size more than 5 mm on ultrasonography were excluded, risk of bias cannot be excluded
Sanawan et al. [5], 2017, Journal of the College of Physicians and Surgeons Pakistan, Pakistan	Randomized controlled study, Level II	150 cases (75 in each group) were randomized into two groups, harmonic scalpel clipless (group A) versus conventional laparoscopic cholecystectomy (group B) with electrocautery group	To determine the efficacy of ultrasound shear in laparoscopic cholecystectomy in terms of intraoperative bleeding	All patients were followed-up for 4 weeks	Intraoperative blood loss in group A was significantly lower than in group B (p = 0.001)	Single Centre, Randomized, power calculation undertaken, no blinding was mentioned, follow up was for 4 weeks only, common bile duct stones, intrahepatic biliary channel dilatations, raised gamma GT or alkaline phosphatase (evidence of obstructive jaundice), fever with rigors and chills, previous hepatobiliary surgery, and previous midline abdominal surgeries were excluded, patients with cystic duct diameter more than 5 mm were excluded, risk of bias cannot be excluded
Awale et al. [6], 2019, World Journal of Laparoscopic Surgery, Nepal	Randomized controlled trial, Level II	112 patients were enrolled into clipless laparoscopic cholecystectomy CLC (53) and conventional cholecystectomy CL (59) groups	To compare fall in haemoglobin between the two groups	Both groups were followed-up for 6 months	The amount of blood loss as demonstrated by the median fall in haemoglobin level was significantly (*p* 0.001) less in the CLC group	Single Centre, randomized, no blinding was mentioned, patients with cholangitis, wide cystic duct >5 mm, CBD stones or dilated CBD, history of jaundice, impaired liver function test, pregnant patients, and suspicion of GB malignancy were excluded, risk of bias cannot be excluded

## 7 Discussion

In 2009, Kandil et al. [2] devised a randomized controlled trial. The study included 140 patients who were randomized into two groups. Group A included 70 patients in whom laparoscopic cholecystectomy was conducted using the traditional method by clipping both cystic duct and artery and dissection of gallbladder from liver bed by diathermy. Group B included 70 patients where laparoscopic cholecystectomy was conducted using harmonic scalpel. Closure and division of both cystic duct and artery and dissection of gallbladder from liver bed by harmonic scalpel. They have found that Intraoperative blood loss was significantly more in the traditional group than in the Harmonic scalpel group (83.31 + 46.23 vs. 43.28 + 31.27; p = 0.0001). The authors concluded that Harmonic scalpel provides a complete haemostasis and is a safe alternative to stander clip of cystic duct and artery.

In 2010, Nakeeb et al. [3] conducted a similar study which included 120 cirrhotic patients where the risk of bleeding is higher than fit and well patients. They found that intraoperative blood loss was significantly greater in the traditional group than in the HS group (133 ± 131.13 vs. 70.13 ± 80.79 ml; p = 0.002). The authors concluded that the Harmonic scalpel provides complete haemostasis and is a safe alternative to the standard cholecystectomy.

Jain et al. [4] in 2011 conducted another randomized controlled trial which included 200 patients and there was a greater fall in haemoglobin (0.53 versus 1.33 g%; P value of 0.001) and haematocrit (1.59 versus 2.60; P value of 0.001) when electrocautery was used compared to Harmonic scalpel.

In 2017, Sanawan et al. [5] conducted a randomized controlled trial which included 150 patients who were randomized into two groups. Half of them underwent clipless cholecystectomy and the other half underwent conventional cholecystectomy. The authors found that Intraoperative blood loss in clipless laparoscopic cholecystectomy was significantly lower than in conventional cholecystectomy group (p = 0.001).

In a recent randomized controlled trial, which was conducted by Awale et al. [6] in 2019. The study included 112 patients who were randomized into two groups comparing clipless laparoscopic cholecystectomy and conventional cholecystectomy. They found that the amount of blood loss as demonstrated by the median fall in haemoglobin level was significantly (*p* 0.001) less in the clipless laparoscopic cholecystectomy group.

The observed limitation to the studies:

1 Risk of bias.
2 Most of the studies excluded particular groups of patients (e.g., >70-year-old and pregnant patients) which might decrease its external validity.

## 8 Clinical bottom line

Five randomized controlled trials proved that intraoperative blood loss is significantly reduced using clipless laparoscopic cholecystectomy. Authors recommend adopting clipless laparoscopic cholecystectomy especially in patients with high risk of intraoperative bleeding.

## Ethical approval

Not Applicable.

## Sources of funding

None.

## Author contribution

SA: devised the idea of the study, conducted literature search and wrote the paper. TA: assisted in literature search and collecting the data. SAA: assisted in literature search and writing the paper. RI: assisted in literature search editing and writing the paper.

## Registration of research studies

1. Name of the registry: not applicable
2. Unique Identifying number or registration ID:
3. Hyperlink to your specific registration (must be publicly accessible and will be checked):

## Guarantor

Sabry Abounozha (SA), Talal Alshahri (TA), Samer Alammari (SAA), Rashid Ibrahim (RI).

## Consent

Not Applicable.

## Declaration of competing interest

None.
